# CircRbfox1 Contributes to Colonic Hypersensitivity in Rats With Diabetes by Altering HuC Subcellular Localization to Regulate RBFOX1 Expression

**DOI:** 10.1002/cns.70896

**Published:** 2026-04-28

**Authors:** Shiyu Zhang, Ji Hu, Chendong Ni, Yilian Zhang, Jiahao Chen, Yajing Liu, Shufen Hu, Fuchao Zhang, Guang‐Yin Xu, Hong‐Hong Zhang

**Affiliations:** ^1^ Department of Endocrinology The Second Affiliated Hospital of Soochow University Suzhou People's Republic of China; ^2^ Clinical Research Center of Neurological Disease The Second Affiliated Hospital of Soochow University Suzhou People's Republic of China; ^3^ Jiangsu Key Laboratory of Neuropsychiatric Diseases and Institute of Neuroscience Soochow University Suzhou P. R. China

**Keywords:** circRbfox1, diabetic colonic hypersensitivity, dorsal root ganglion, HuC, RBFOX1

## Abstract

**Aims:**

The purpose of this study was to explore the molecular mechanism of circRNA mediated colonic hypersensitivity in rats with diabetes.

**Methods:**

Intraperitoneal injection of streptozocin female rats was used to induce Type 1 Diabetes Mellitus. A combination of molecular biology and behavioral approaches was used to investigate the role of circRbfox1 in the pathogenesis of colonic hypersensitivity in diabetic rats.

**Results:**

A new circular RNA‐Rbfox1, derived from the host gene encoding RNA‐Binding Protein Fox1, as a mechanism of visceral pain is identified. We confirmed that circRbfox1 remarkably upregulated in T13‐L2 dorsal root ganglions in rats with diabetes, and could interact with the RNA‐binding protein HuC to promote its transition from the cytosol into the nucleus to promote the translation level of RBFOX1. In addition, the increased expression of Rbfox1 could further promote colonic hypersensitivity in diabetic rats by regulating the expression of Cav1.3.

**Conclusions:**

We conclude that circRbfox1 serves as pain regulator in diabetic colonic hypersensitivity and provides a potential therapeutic target in clinic.

## Graphical abstract

circRbfox1 mediates diabetic colonic hypersensitivity by binding the RNA‐binding protein HuC, facilitating its nuclear translocation in T13‐L2 DRGs. This enhances transcription of the parent gene *Rbfox1*, culminating in increased expression of the L‐type calcium channel Cav1.3. Collectively, these data reveal a previously unrecognized circRNA‐dependent pathway and highlight a potential target for treating diabetic neuropathic pain.

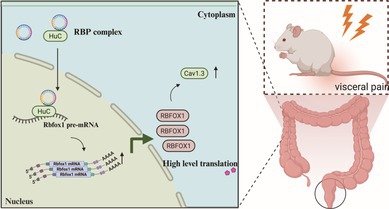

**Abbreviations**
AWR: Abdominal Withdrawal Reflex
circRNA: circular RNA
CON: Control
CRD: Colorectal Distention
DM: Diabetes mellitus
FISH: Fluorescence in situ hybridization
LV: Lentivirus
NC: Negative Control
RIP: RNA‐protein immunoprecipitation
SD: Sprague–Dawley
STZ: streptozocin
T13‐L2 DRGs: T13‐L2 dorsal root ganglions
T1DM: Type 1 Diabetes Mellitus

## 1 Introduction

Diabetes mellitus (DM) is one of the fastest‐growing metabolic disorders characterized by chronic hyperglycemia. It is estimated that in 2045 there will be 693 million adults who suffer from diabetes globally [1]. The pervasive influence of sustained hyperglycemia extends its reach into nearly every bodily tissue, triggering a cascade of complications affecting multiple systems. A noteworthy observation from previous research reveals that over 50% of individuals with diabetes experience a spectrum of gastrointestinal symptoms, encompassing early satiety, diminished appetite, nausea, vomiting, abdominal pain, diarrhea, and constipation [2]. Diabetic visceral discomfort is one of these symptoms that is frequently seen in clinic. Currently, a thorough knowledge of the pathogenesis of diabetic visceral pain remains elusive.

The persistent diabetic condition leads to an array of gastrointestinal disorders by impairment of the autonomic nervous systems, including the sympathetic and parasympathetic nervous systems [3]. Notably, diabetic visceral pain emerges as a critical manifestation of autonomic nerve dysfunction within the gastrointestinal tract. Sensory neurons provide sensory information to the dorsal horn of the spinal cord via their nerve terminals, which are dispersed throughout the periphery. Humans have a standard 31 pairs of spinal nerves (C1‐Co1), with only 12 thoracic (T1‐T12) segments. In rats, the distal colon is primarily innervated by sensory neurons located in the T13‐L2 DRGs (a nomenclature specific to rodent anatomy). The pivotal involvement of T13‐L2 DRGs assumes a fundamental role in the genesis of diabetic colonic hypersensitivity.

CircRNAs are a new kind of non‐coding RNAs that are generated from back‐splicing of pre‐mRNAs to form covalently closed transcripts without a terminal 5'cap and a 3'polyadenylated tail [4]. CircRNAs exhibit higher stability than their linear counterparts because of the distinction in their loop structure. Multiple studies have reported that circRNAs play a variety of regulatory functions in diverse diseases, including cardiovascular, nervous system, endocrine, and neoplastic disorders. For instance, circRNAs can control the transcription and splicing processes [5]. Additionally, because it has numerous microRNA binding sites, it can operate as a “microRNA sponge” and control the function of microRNAs on its target genes [6]. Moreover, some circRNAs contain IRES elements, which allow them to encode short peptides with biological functions [7]. In addition, circRNAs can also interact with multiple proteins to regulate their target downstream genes [8, 9, 10]. Previous research has proven that circHIPK3 is involved in the occurrence of neuropathic pain in diabetic rats [11]. Here, we aimed to identify the functions of circRNAs in inducing visceral pain in diabetes.

In this study, we sought to discover roles played by circRNAs in regulating diabetic colonic hypersensitivity. We identified a circRNA derived from Rbfox1 pre‐mRNA that we call circRbfox1. We found that streptozocin (STZ)‐induced diabetes led to an increased expression of circRbfox1 in T13‐L2 DRGs in rats with diabetes. circRbfox1 could interact with the RNA‐binding protein HuC, facilitating its translocation from the cytoplasm to the nucleus and, consequently, enhancing the transcriptional activity of RBFOX1. Moreover, the elevated levels of RBFOX1 are anticipated to stimulate the expression of Cav1.3 within T13‐L2 DRGs of diabetic rats, thereby exacerbating colonic hypersensitivity.

## 2 Methods

### 2.1 Generation of Streptozotocin‐Induced Diabetes

Adult female Sprague–Dawley (SD) rats (weighing 180‐200 g) were housed 4 per cage in a temperature‐controlled (25°C ± 1°C) and 12 h/12 h light/dark cycle room. The reason that we choose female rats as our modeling animal is to minimize the variation between male and female rats since early studies have reported the gender differences in pain threshold both in human [12] and in animal model [13].

DM model was induced by a single intraperitoneal injection of STZ (75 mg/kg, Sigma Chemicals, USA), which was freshly dissolved in citrate buffer (10 mmol/L, Na citrate, pH = 4.3–4.4), as described previously [14, 15]. The rat control (CON) only received an equivalent volume of citrate buffer. Fasting blood glucose concentration obtained from the tail vein was measured by glucometer (Johnson & Johnson, USA). Only rats with blood glucose concentrations higher than 15.0 mmol/L were further used in the study and data analysis (Figure S1).

### 2.2 Western Blotting

The total protein was separated by SDS‐PAGE electrophoresis and transferred onto PVDF membrane (Millipore, USA). After incubation with 5% non‐fat milk for 2 h to block nonspecific binding, the membranes were immunoblotted overnight at 4°C with the following primary antibodies: anti‐HuC (1:1000; Thermo, USA), anti‐RBFOX1 (1:5000; Abcam, USA), anti‐Cav1.3 (1:200; Alomone, Israel), anti‐GAPDH (1:1000; Goodhere, China), and anti‐histone3 (1:1000; Millipore, Germany). Then, the membranes were incubated with the secondary antibodies for 2 h at room temperature. Immunoreactive proteins were detected by enhanced chemiluminescence (ECL kit; GE Healthcare Pharmacia Biotech). The densities of protein bands were analyzed with NIH Image software. For all Western blot analyses, the most representative images are shown in this article.

### 2.3 RNA Extraction and Quantitative Real‐Time PCR

Total RNA was isolated by TRIzol reagent (Invitrogen, USA). cDNA was synthesized using EasyScript First‐Strand cDNA Synthesis SuperMix kit (Transgen Biotech, China). qPCR SYBR Green master mix (Yeasen Biotechnology, China) was used for quantitative real‐time PCR. The primer sequences used in this study are shown in Table 1.

**TABLE 1:** Primers used in the study.

Primers	Sequence (5′ → 3′)
Gapdh‐F	TGGAGTCTACTGGCGTCTT
Gapdh‐R	TGTCATATTTCTCGTGGTTCA
Rbfox1‐F	CCTTATGCCTCAGCGCAGTT
Rbfox1‐R	TGGTCTGGCCGGTGTACTCT
CircRNA‐Rbfox1‐F	GTGCCATGTGCCTGGCTTTC
CircRNA‐Rbfox1‐R	TTGGGGGTGGGGGCGCCGCA
Cav1.3‐F	CTGCGATGTGCCAGTAGGT
Cav1.3‐R	GTGGTGGTTGATGAGTTTGTG
U6‐F	CTCGCTTCGGCAGCACATA
U6‐R	TGGAACGCTTCACGAATTTG
HuC‐F	GCTGCTGAGCCTATCACGGT
HuC‐R	AGAGGACTGGTACAGGTGGGTG

### 2.4 RNase R Digestion

For RNase R digestion, 1 μg of total RNA was incubated for 30 min at 37°C with or without 2.5 U of RNase R (Epicenter Technologies, Madison, WI). Then, the treated RNAs were reverse transcribed with specific primers and detected by a real‐time PCR assay.

### 2.5 CRD and Behavioral Testing

Colonic hypersensitivity was assessed by grading the behavioral response of rats to Graded Colorectal Distention (CRD) at the age of 4 weeks based on previous publications [16]. Abdominal Withdrawal Reflex (AWR) score was used to observe the animal response to graded CRD (20, 40, 60, and 80 mmHg) and the assignment of an AWR score: 0, no behavioral response to CRD; 1, brief head movement followed by immobility; 2, contraction of abdominal muscles; 3, lifting of abdomen; 4, body arching and lifting of pelvic structures. Each pressure was repeated three times and the average value was used to reflect the visceral hypersensitivity. All behavioral tests were performed in a blinded manner.

### 2.6 Fluorescence in Situ Hybridization (FISH)

The digoxigenin‐labeled miRCURY LNA miRNA detection probe for circRbfox1 was designed and synthesized by Qiagen (Germany). IsHybridIn Hybridization (ISH) Kit (Biochain, USA) was used to detect the probe signals of circRbfox1. Experiments were conducted using ISH Kit based on the manufacturer's manual with minor modifications as described previously.

### 2.7 Immunofluorescence Study

Rats were put under deep anesthesia and then perfused with saline and 4% paraformaldehyde to fix. T13‐L2 DRGs were removed, post‐fixed, and dehydrated. DRGs were cut into 15 μm sections. Sections were treated with 0.5% Triton X‐100 and immunoblotted overnight at 4°C with the following primary antibodies: anti‐HuC (1:100; Thermo, USA), anti‐RBFOX1(1:250; Abcam, USA), anti‐Cav1.3(1:100; Alomone, Israel), anti‐NeuN (1:100; Millipore, Germany), anti‐IB4(1:200; Sigma, Germany), anti‐GCRP (1:100; Invitrogen, USA). Then, the sections were incubated with secondary antibodies i.e., Alexa Fluor488/555/405(1:1000; Invitrogen, USA).

### 2.8 Cytosolic/Nuclear Fractionation

The subcellular location of circRbfox1 was detected by Cytosolic/Nuclear Fractionation kit (Beyotime, China) as manufacturer's manual with minor modifications. RNase inhibitor (Beyotime, China) was used to prevent RNA degeneration. PMSF (Beyotime, China)was used to prevent protein degeneration.

### 2.9 RNA‐Protein Immunoprecipitation (RIP)

Magna RIP RNA‐Binding Protein Immunoprecipitation Kit (Millipore, USA) was used to detect the interaction between circRbfox1 and HuC. Briefly, Anti‐IgG and Anti‐HuC were incubated with magnetic beads at room temperature for 30 min. Then the antibody‐beads complexes were incubated with T13‐L2 DRGs lysis at 4°C overnight. The RNA‐beads complexes were eluted in eluted buffer at 55°C for 30 min. Target RNA was extracted and detected by RT‐PCR.

### 2.10 Cell Transfection and Lentiviral Vector Transduction

For *circRbfox1* knockdown, a circRbfox1 shRNA lentivirus or a negative control lentivirus was used (GENESEED). For *HuC* knockdown, a HuC shRNA lentivirus or a negative control lentivirus was used (DongLing Biotech). For RBFOX1 knockdown, an RBFOX1 shRNA lentivirus or a negative control lentivirus was used (DONGLNG BIOTECH). All lentivirus (10 μL) was administered by one intrathecal injection into rats. HuC overexpression plasmid (GENE Pharma) was transfected into neuronal ND7/23 cells by Lipofectamine 2000 (Invitrogen, USA) following the manufacturer's instruction*s*. Cell RNA and protein were obtained at least 48 h after transfection.

### 2.11 Statistical Analysis

Statistical analysis was conducted using Prism 6 (GraphPad) software. The experimental data were expressed as mean ± SEM. The differences between groups were compared using Student's *t*‐test or two‐way repeated measures ANOVA followed by Tukey post hoc test. A *p‐*value of < 0.05 was considered statistically significant.

## 3 Results

### 3.1 Identification and Characteristics of circRbfox1 in T13‐L2 DRGs in Rats

**FIGURE 1:**
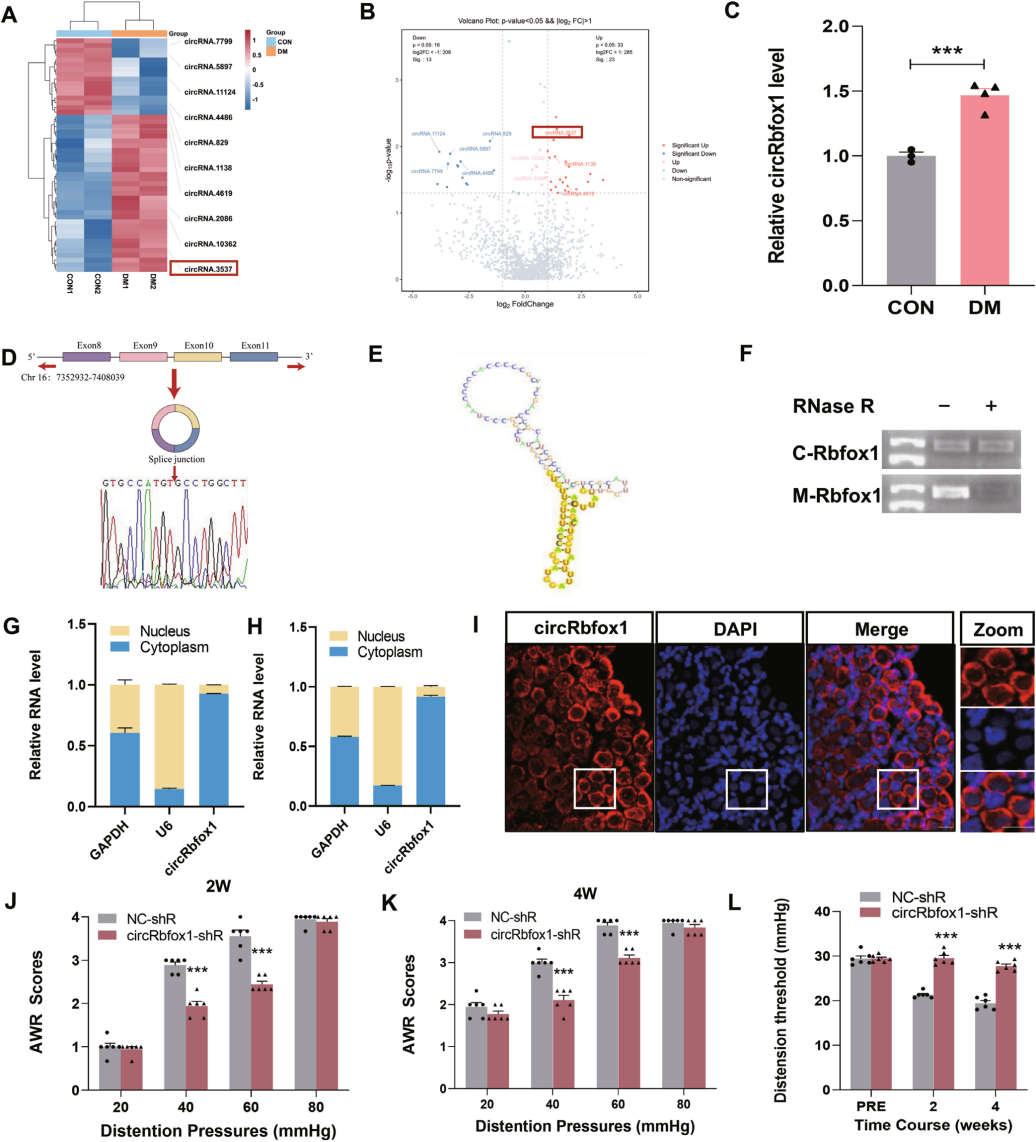
Identification and characteristics of circRbfox1 in T13‐L2 DRGs in rats. (A) A heatmap showing circRNA expressions in the DRGs of diabetic mice, with red and blue colors denoting upregulation and downregulation, respectively. Differential expression was defined as a fold change of ≥ 2 and *p < 0.05. The red box highlights circRbfox1. (B) Volcano plots showed circRNA expressions in the DRGs of diabetic mice, with red and blue colors denoting upregulation and downregulation, respectively. (C) RT‐qPCR validation of significantly increased circRbfox1 expression in T13‐L2 DRGs of diabetic rats (CON: Control group, *n* = 3; DM: Diabetic group, *n* = 4; ***p < 0.001, two‐sample *t*‐test). (D) Sequencing analysis of head‐to‐tail splicing junction in circRbfox1. (E) Secondary structure of functional sequences within circRbfox1. (F) Comparison of circRbfox1 and Rbfox1 mRNA expression levels by RNase R digestion analysis; (C‐Rbfox1: CircRNA‐Rbfox1, M‐Rbfox1: mRNA‐Rbfox1). (G, H) RT‐qPCR analysis of circRbfox1's subcellular distribution in T13‐L2 DRGs of control and diabetic rats (G: CON rats, H: DM rats; *n* = 3 for each group). U6 served as a nuclear positive control, and GAPDH as a cytoplasmic positive control. (I) FISH assay identifying the subcellular location of circRbfox1 in T13‐L2 DRGs of control rats. Scale bar = 50 μm. (J, K) abdominal withdrawal reflex (AWR) was recorded as colonic sensitivity at 2 weeks and 4 weeks after injection of circRbfox1 shRNA lentivirus. (L) CRD was used to grade the behavioral response of rats by assessing colonic hypersensitivity after injection of circRbfox1 shRNA lentivirus. (NC‐shR: Negative control shRNA, circRbfox1‐shR: CircRbfox1‐shRNA, *n* = 6 in each group, PRE: Before LV injection and post successful modeling, ***p < 0.001, two‐way repeated‐measures ANOVA followed by Tukey post hoc test).

To identify potential circRNA candidates that regulate the progression of diabetic colonic hypersensitivity, we used high‐throughput sequencing to profile differentially expressed circRNAs in DRGs in wild type and Akita mice. With an integrated analysis, we identified circRNA.3537, a novel RNA, that was significantly upregulated in DRGs of diabetic mice and had the largest difference in expression compared to the control group (Figure 1A–C). circRNA.3537 is derived from Rbfox1 pre‐mRNA, that we call circRbfox1 according to our sequencing dates. circRbfox1, formed by circularization of exons 8–11 of the gene Rbfox1, was located at chromosome 16:7352932–7,408,039 (Figure 1D). Sanger sequencing was performed to verify the back‐splicing junction site of circRbfox1. We predicted and found through informatics that circRbfox1 lacks open reading frames, polyadenylation sites, ribosome binding sites, and terminators independent of Rho, indicating that it is unlikely to synthesize peptides. However, circRbfox1 contains a functional RNA sequence located at 133–174 which is 42 bp long and has the function of binding to the target gene (Figure 1E). Moreover, resistance to RNase R digestion assay confirmed that circRbfox1 existed as a closed‐loop structure (Figure 1F).

After fractionating T13‐L2 DRGs tissues into nuclear and cytoplasmic lysates, RT‐qPCR analysis showed that the majority of the circRbfox1 was located in the cytoplasm, with a minor quantity dispersed throughout the nucleus (Figure 1G,H). FISH showed that circRbfox1 was localized in both the nucleus and cytoplasm, which is consistent with the observations in fractionated lysates of T13‐L2 DRGs (Figure 1I).

### 3.2 circRbfox1 Contributes to Colonic Hypersensitivity in Rats With Diabetes

To determine whether circRbfox1 plays a role in the development of diabetic colonic hypersensitivity, we successfully transduced circRbfox1 shRNA lentivirus (LV‐circRbfox1 shRNA) to silence the circRbfox1 gene. Colonic sensitivity was assessed by monitoring the AWR in response to CRD. Our findings revealed that a single intrathecal injection of LV‐circRbfox1 shRNA led to a significant reduction in colonic hypersensitivity in diabetic rats after 2 weeks, in comparison to the group receiving the negative control shRNA (NC shRNA) injection. Notably, this effect appeared to persist for at least 4 weeks of observation (Figure 1J–L).

### 3.3 circRbfox1 Contributes to Colonic Hypersensitivity by Interacting With RNA Binding Protein HuC

**FIGURE 2:**
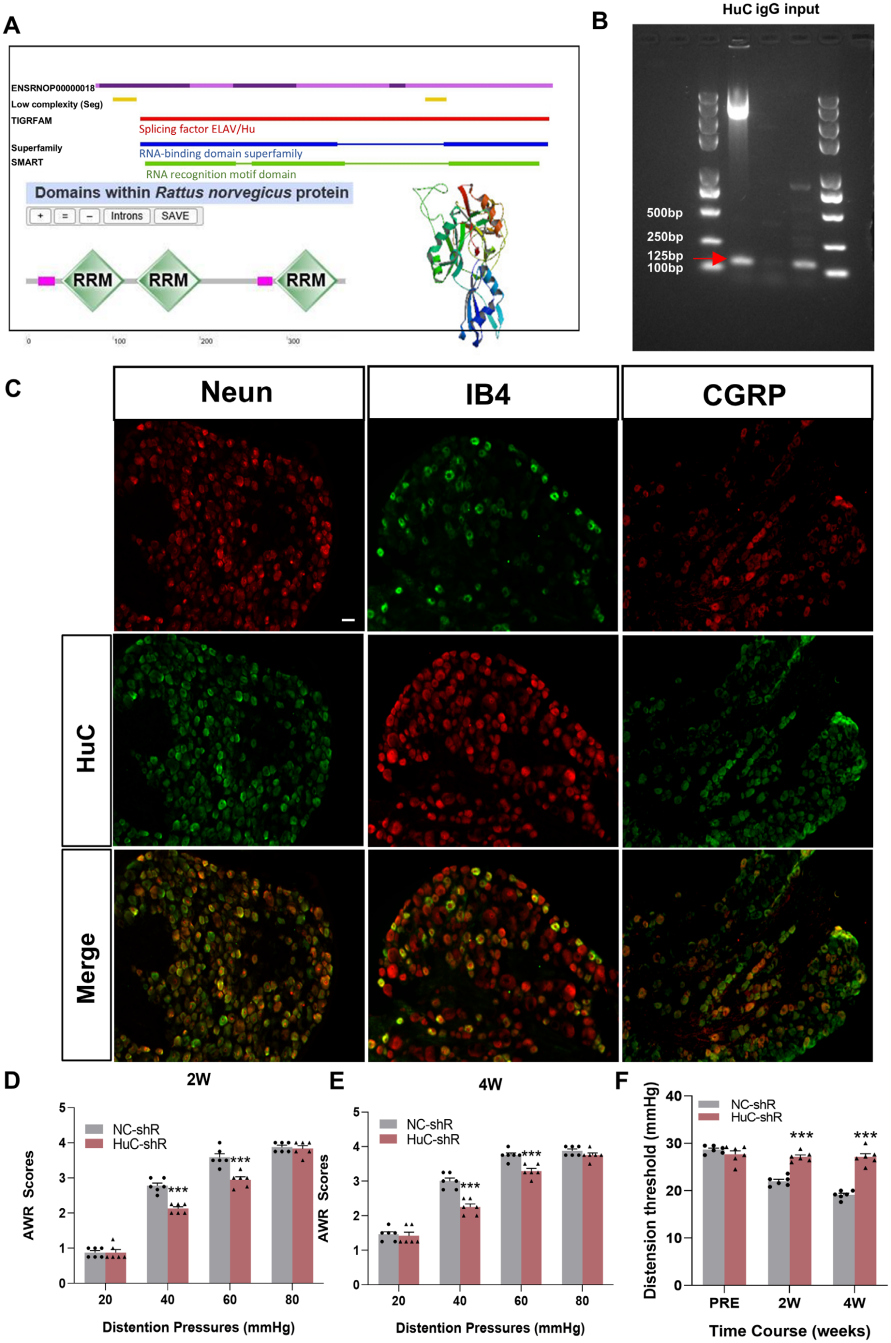
circRbfox1 contribute to colonic hypersensitivity by interacting with HuC. (A) Three RNA recognition domains of HuC. (B) RIP results using anti‐HuC and normal rabbit IgG as immunoprecipitation antibodies. Lane 1 indicates HuC enrichment of circRbfox1 (approximately 125 bp), lane 2 shows no significant bands with IgG, indicating that normal rabbit IgG did not enrich circRbfox1, and lane 3 represents the Input, where specific circRbfox1 cDNA is observable. (C) Immunofluorescence results indicate co‐localization of HuC primarily with neurons labeled with NeuN, as well as with small to medium‐sized peptide‐neurons labeled with CGRP, and small to medium‐sized non‐peptidergic neurons labeled with IB4 in control rats of T13‐L2 DRGs. Scale bar = 50 μm. (D, E) AWR scores of diabetic rats at 2 weeks and 4 weeks after the injection of HuC shRNA lentivirus. (F) The distension thresholds of diabetic rats at 2 weeks and 4 weeks after the injection of HuC shRNA lentivirus (NC‐shR: Negative control shRNA, HuC‐shR: HuC‐shRNA, *n* = 6 for each group, PRE: Before LV injection and post successful modeling, ***p < 0.001, two‐way repeated‐measures ANOVA followed by Tukey post hoc test).

To better map the role of circRbfox1 in colonic hypersensitivity in diabetic rats, we predicted and found that the RNA‐binding protein HuC can interact with the two RNA spliceosomes of Rbfox1 pre‐mRNA, which are circRbfox1 and its parent gene Rbfox1 mRNA. HuC has three RRMs (located at 40–113, 126–201, 285–358) (Figure 2A). Therefore, we conducted a RIP assay to validate the binding of HuC to circRbfox1. As shown in Figure 2B, circRbfox1‐specific bands could be precipitated in the anti‐HuC antibody and input group, but not the IgG group. The result suggests that circRbfox1 may interact with HuC. In addition, we performed immunohistochemistry to determine the location of HuC. As shown in Figure 2C, most HuC was co‐localized with neurons labeled with NeuN, as well as with small to medium‐sized peptide‐neurons labeled with CGRP, and small to medium‐sized non‐peptidergic neurons labeled with IB4 in normal rat T13‐L2 DRGs. To determine whether HuC plays a role in the development of diabetic colonic hypersensitivity, we successfully transduced HuC shRNA lentivirus (LV‐HuC shRNA) to silence the HuC gene. AWR responding to CRD was recorded as colonic sensitivity. The results showed that a single intrathecal injection of LV‐HuC shRNA significantly alleviated colonic hypersensitivity in diabetic rats after 2 weeks as compared to injection of the NC shRNA group, and this effect may have persisted at least through the 4th week of observation (Figure 2D–F).

### 3.4 circRbfox1 Could Change Subcellular Localization of HuC in Diabetic Conditions

**FIGURE 3:**
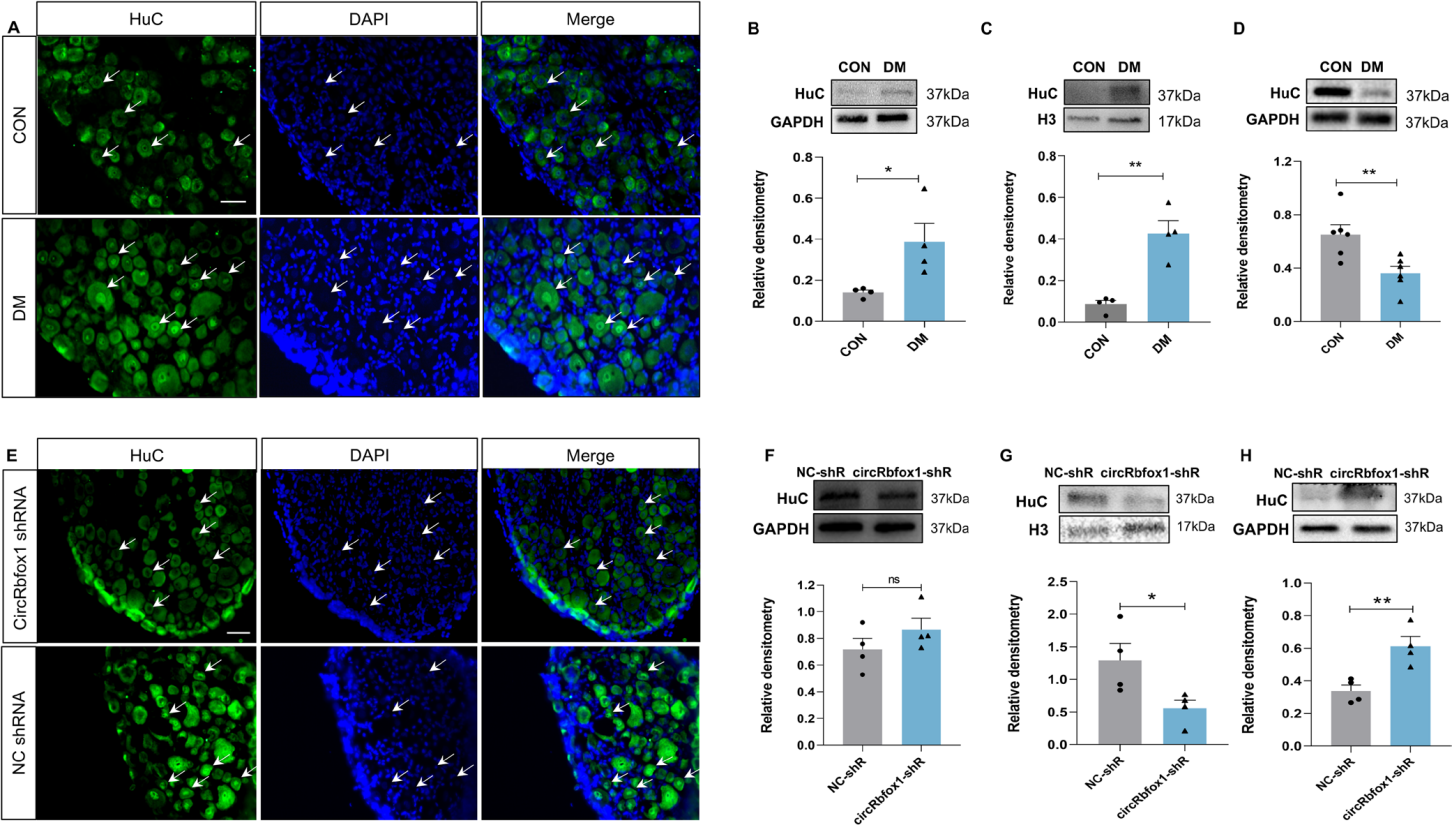
circRbfox1 could change subcellular localization of HuC in diabetic conditions. (A) Immunofluorescence staining confirmed the fluorescence intensity of HuC within the nucleus and cytoplasm in CON and DM rats. Scale bar = 50 μm. (B) Western blotting detected the whole‐cell expression of HuC in T13‐L2 DRGs (CON: *N* = 4, DM: *N* = 4, *p < 0.05, two‐sample *t*‐test). (C) Western blotting detected the nucleus expression of HuC in T13‐L2 DRGs (CON: *N* = 4, DM: *N* = 4, **p < 0.01, two‐sample *t*‐test). (D) Western blotting detected the expression of HuC in the cytoplasm in T13‐L2 DRGs (CON: *N* = 6, DM: *N* = 6, **p < 0.01, two‐sample *t*‐test). (E) Immunofluorescence staining revealed a significant reduction in the fluorescence intensity of HuC in the cell nuclei of T13‐L2 DRGs after intrathecal injection of circRbfox1 shRNA lentivirus compared to NC group rats. Scale bar = 50 μm. (F) In comparison to NC group, intrathecal injection of circRbfox1 shRNA lentivirus in DM rats did not alter the overall cellular expression of HuC in T13‐L2 DRGs (NC‐shR: Negative control shRNA, circRbfox1‐shR: CircRbfox1‐shRNA, *n* = 4 for each group, *p* > 0.05, ns: No significant, two‐sample *t*‐test). (G) In contrast to NC group, intrathecal injection of circRbfox1 shRNA lentivirus in DM rats resulted in a reduction in the expression of HuC in the cell nuclei of T13‐L2 DRGs (NC‐shR: Negative control shRNA, circRbfox1‐shR: CircRbfox1‐shRNA, *n* = 4 for each group, *p < 0.05, two‐sample *t*‐test). (H) In comparison to NC group, intrathecal injection of circRbfox1 shRNA lentivirus in DM rats led to an elevation in the expression of HuC in the cytoplasm of T13‐L2 DRGs (NC‐shR: Negative control shRNA, circRbfox1‐shR: CircRbfox1‐shRNA, *n* = 4 for each group, **p < 0.01, two‐sample *t*‐test).

To further verify the specific role of HuC in colonic hypersensitivity in rats with diabetes, we first performed immunofluorescence staining. The results showed that a substantial increase in the fluorescence intensity of HuC within the nucleus of T13‐L2 DRGs in diabetic rats compared with CON rats (Figure 3A). Additionally, we examined the whole‐cell expression of HuC and observed a significant elevation in HuC levels within the T13‐L2 DRGs in diabetic rats (Figure 3B). Subsequently, we isolated nuclear and cytoplasmic fractions from both CON and diabetic rats. The analysis demonstrated a marked upregulation of HuC expression in the nucleus of T13‐L2 DRGs in diabetic rats in comparison to the CON group, whereas the expression in the cytoplasm was significantly reduced (Figure 3C,D).

Recent investigations have substantiated the capacity of certain circRNAs to influence the positioning of RNA‐binding proteins to regulate downstream target proteins. We wonder if the subcellular change of HuC is mediated by circRbfox1. To prove it, we first detected the fluorescence intensity of HuC in the nucleus and cytoplasm after intrathecal injection of LV‐circRbfox1 shRNA and LV‐NC shRNA in rats with diabetes, respectively. The results indicate that intrathecal injection of LV‐circRbfox1 shRNA led to a pronounced reduction in the fluorescence intensity of HuC within the nucleus (Figure 3E), supporting our hypothesis. In line with the observations above, the overall cellular protein expression of HuC in T13‐L2 DRGs in rats with diabetes after intrathecal injection of LV‐circRbfox1 shRNA remained unchanged (Figure 3F). However, the expression of nuclear protein decreased while cytoplasmic protein expression increased (Figure 3G,H). Taken together, these observations provide evidence that circRbfox1 has the capacity to modulate the subcellular localization of HuC.

### 3.5 HuC Contributes to Colonic Hypersensitivity by Regulating RBFOX1 Expression in Rats With Diabetes

**FIGURE 4:**
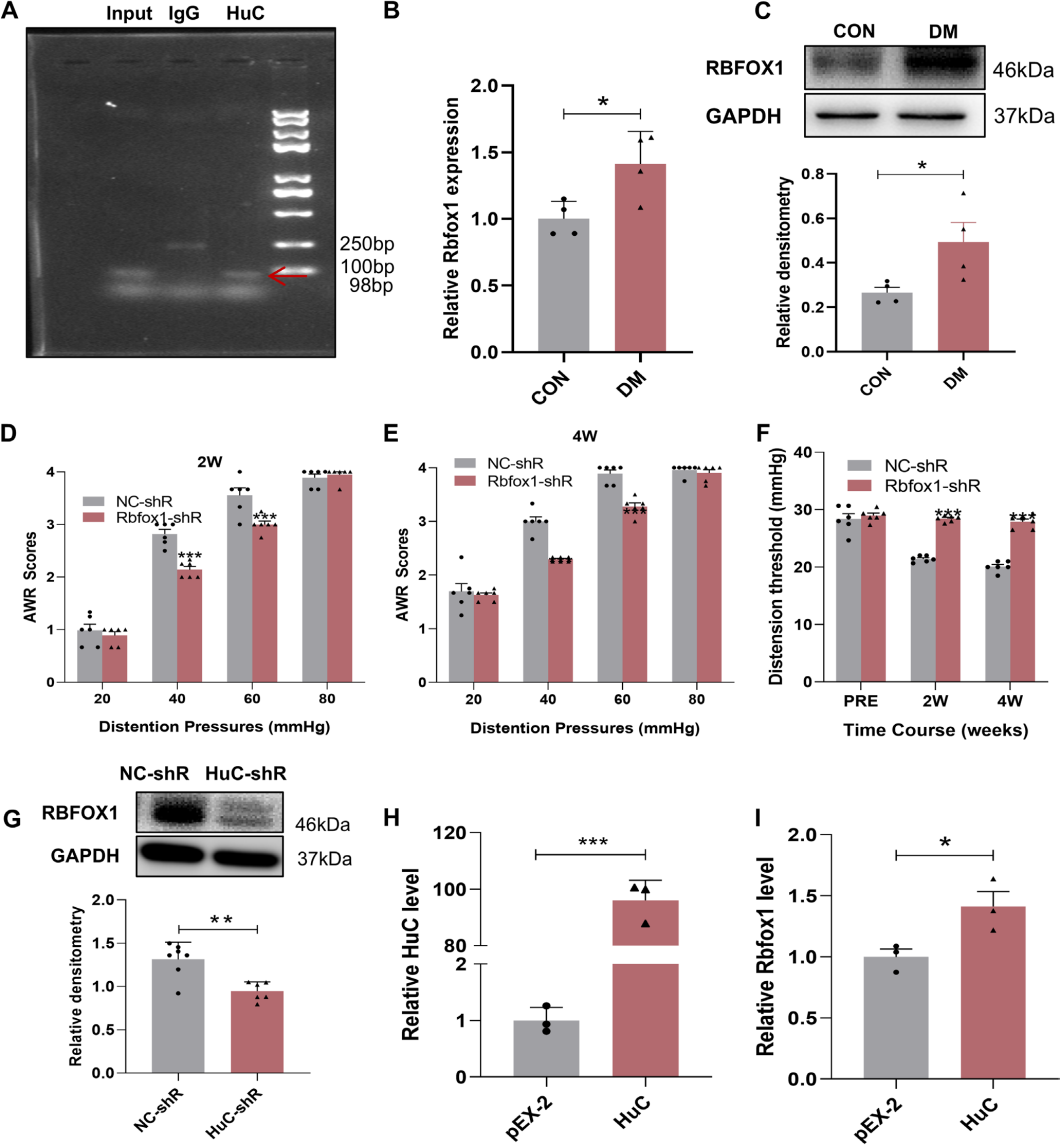
HuC contributes to colonic hypersensitivity by regulating RBFOX1 in rats with diabetes. (A) RIP results using anti‐HuC and normal rabbit IgG as immunoprecipitation antibodies. Lane 1 represents the Input enrichment of Rbfox1 (approximately 98 bp), lane 2 shows no significant bands with IgG, indicating that normal rabbit IgG did not enrich Rbfox1, and lane 3 using HuC antibody, where specific Rbfox1 cDNA is observable. (B) Compared to CON group, the Rbfox1 mRNA level was greatly improved in T13‐L2 DRGs of DM rats (CON: Control group, *n* = 4; DM: Diabetic group, *n* = 4; *p < 0.05, two‐sample *t*‐test). (C) Compared to CON group, the protein level of RBFOX1 was greatly improved in T13‐L2 DRGs of DM rats (CON: Control group, *n* = 4; DM: Diabetic group, *n* = 4; *p < 0.05, two‐sample *t*‐test). (D, E) AWR scores of diabetic rats at 2 weeks and 4 weeks after the injection of HuC shRNA lentivirus. (F) The distension thresholds of diabetic rats at 2 weeks and 4 weeks after the injection of HuC shRNA lentivirus (NC‐shR: Negative control shRNA, Rbfox1‐shR: Rbfox1‐shRNA, *n* = 6 for each group, PRE: Before LV injection post successful modeling, ***p < 0.001, two‐way repeated‐measures ANOVA followed by Tukey post hoc test). (G) Compared to NC group, intrathecal injection of HuC shRNA lentivirus in DM rats resulted in a reduction in the expression of RBFOX1 of T13‐L2 DRGs (NC‐shR: Negative control shRNA, *n* = 7, circRbfox1‐shR: CircRbfox1‐shRNA, *n* = 6, **p < 0.01, two‐sample *t*‐test). (H, I) In vitro transfection of HuC overexpressing plasmid showed increased mRNA levels of Rbfox1 mRNA (pEX‐2 group: No‐load plasmid group, *n* = 3, HuC group: HuC overexpression plasmid, *n* = 3, *p < 0.05, ***p < 0.001, two sample *t*‐test).

To further confirm the role of circRbfox1 in diabetic colonic hypersensitivity, we assessed the expression of its parental gene, Rbfox1. RIP assays performed against Rbfox1 mRNA confirmed its interaction with HuC (Figure 4A). The expression of Rbfox1 both at the mRNA and protein level was significantly enhanced in T13‐L2 DRGs 4 weeks after STZ injection when compared with age‐ and sex‐matched control rats (Figure 4B,C). Based on these findings, we explored the impact of Rbfox1 on the colonic hypersensitivity of diabetic rats. A single intraperitoneal injection of LV‐Rbfox1 shRNA resulted in a substantial attenuation of AWR scores and an increase in CRD in diabetic rats when compared to the LV‐NC shRNA group during the 2nd and 4th week after STZ injection (Figure 4D–F). Collectively, these data strongly implicate the significant role of RBFOX1 in colonic hypersensitivity in rats with diabetes. Next, we found that the expression level of RBFOX1 protein was significantly reduced after a single intrathecal injection of LV‐HuC shRNA compared to the LV‐NC shRNA group in diabetic rats (Figure 4G). In addition, we transfected HuC overexpressed plasmids in ND7/23 cells and found that the mRNA levels of Rbfox1 were significantly increased (Figure 4H,I). Therefore, we assumed that HuC plays a pivotal role in the regulation of RBFOX1 expression.

### 3.6 circRbfox1 May Regulate the Expression of RBFOX1 by Changing the Subcellular Localization of HuC

**FIGURE 5:**
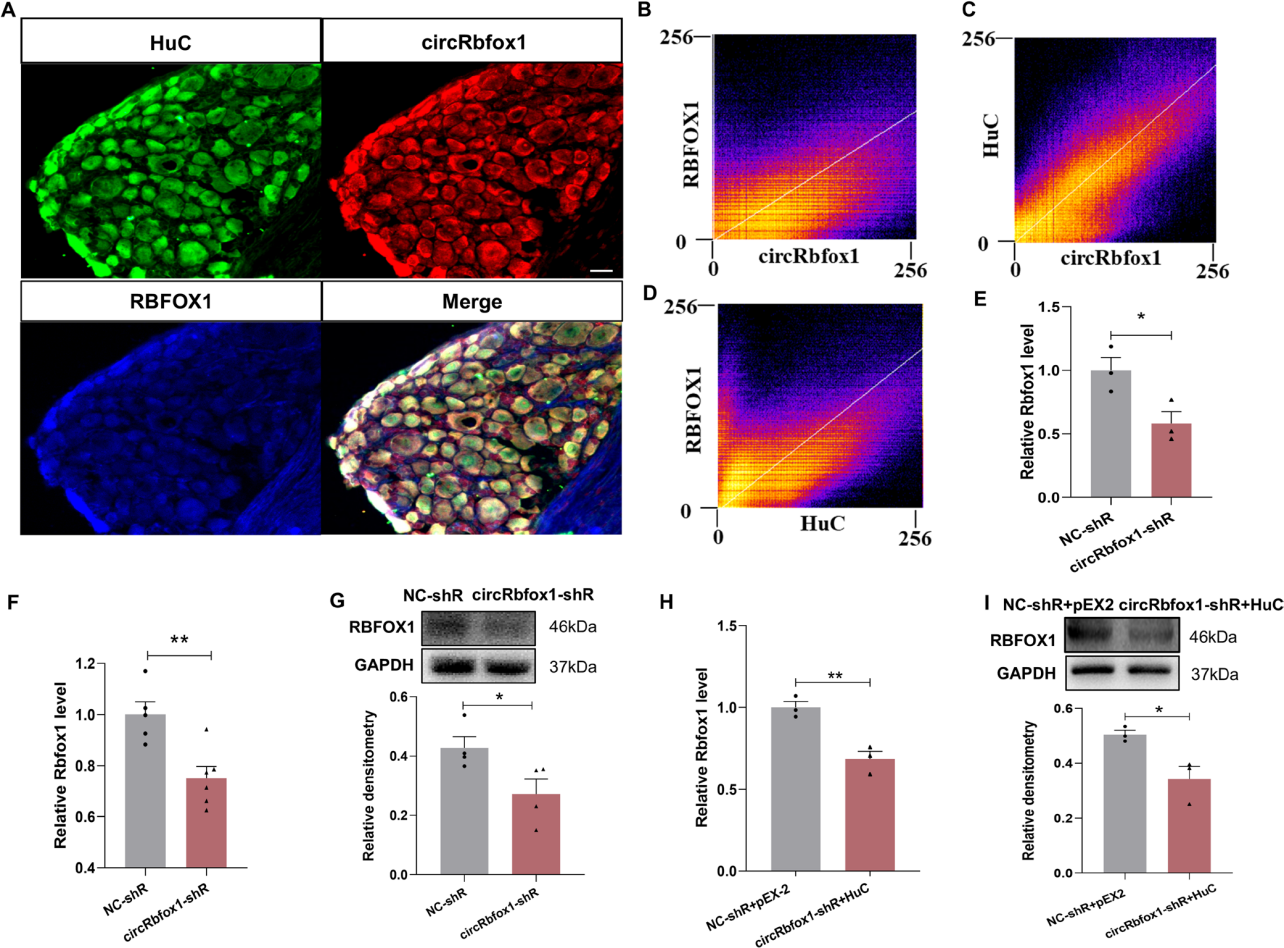
circRbfox1 may regulate the expression of RBFOX1 by changing the subcellular localization of HuC. (A) The colocalization of circRbfox1, HuC and RBFOX1 in control rats of T13‐L2 DRGs was detected using a FISH compared with IF assay. The circRbfox1 probe was labeled with Cy3 (red), the HuC was stained with green, and RBFOX1 were stained with blue. Scale bar =50 μm. (B‐D) Analysis of the Pearson's correlation coefficients demonstrated co‐localized expression among circRbfox1, HuC, and RBFOX1. Pearson's correlation analysis (unthresholded) yielded R values of 0.87, 0.64, and 0.69. (E) The Rbfox1 mRNA level was significantly decreased after transfected with circRbfox1 shRNA lentivirus in vitro (NC‐shR: Negative control shRNA，circRbfox1‐shR: CircRNA‐Rbfox1‐shRNA, *n* = 3 for each group, *p < 0.05, two sample *t*‐test). (F) The level of Rbfox1 mRNA was greatly reduced in DM rats after injection of circRbfox1‐shR lentivirus compared to NC‐shR group in vivo (NC‐shR: Negative control shRNA, *n* = 5, circRbfox1‐shR:CircRbfox1‐shRNA, *n* = 6，**p < 0.01, two sample *t*‐test). (G) In comparison to NC‐shR group, intrathecal injection of circRbfox1 shRNA lentivirus in DM rats greatly reduced the protein level of RBFOX1 in T13‐L2 DRGs in vivo (NC‐shR: Negative control shRNA, *n* = 4, circRbfox1‐shR:CircRNA‐Rbfox1‐shRNA, *n* = 4，*p < 0.05, two sample *t*‐test). (H) In vitro co‐transfection of circRbfox1 shRNA lentivirus and HuC overexpression plasmid, the mRNA level of Rbfox1 was significantly reduced compared with the CON group (NC‐shR + pEX‐2: *N* = 3, circRbfox1‐shR + HuC: *N* = 3, **p < 0.01, two sample *t*‐test). (I) In vitro co‐transfection of circRbfox1 shRNA lentivirus and HuC overexpression plasmid resulted in a significant decrease in RBFOX1 protein levels compared with the control group (NC‐shR + pEX‐2: *N* = 3, circRbfox1‐shR + HuC: *N* = 3, *p < 0.05, two sample *t*‐test).

In our initial investigation, we established that circRbfox1 potentially facilitates the nuclear translocation of HuC, while also recognizing the involvement of Rbfox1, the parent gene of circRbfox1, in the manifestation of diabetic colonic hypersensitivity. We postulated the presence of a regulatory nexus among these elements. To delve deeper, FISH combined with immunofluorescence was performed to assess the co‐localization between circRbfox1, HuC, and RBFOX1. The results showed that circRbfox1, HuC, and RBFOX1 were co‐expressed in T13‐L2 DRGs of CON rats (Figure 5A). Figure 5B–D show the analysis of Pearson's correlation coefficients of circRbfox1, HuC, and RBFOX1. The aforementioned results prompted us to study whether circRbfox1 exerts regulatory control over RBFOX1 expression. We detected a significant decrease in the mRNA level of Rbfox1 in ND7/23 cells transfected with LV‐circRbfox1 shRNA (Figure 5E). More importantly, we found that the mRNA and protein levels of RBFOX1 were significantly reduced after a single intrathecal injection of LV‐circRbfox1 shRNA in rats with diabetes (Figure 5F,G). All results above proved that circRbfox1 may regulate the expression of RBFOX1 in T13‐L2 DRGs of rats with diabetes.

To confirm the regulatory influence of circRbfox1 and HuC as individual factors on Rbfox1, we carried out separate experiments. Remarkably, when ND7/23 cells were co‐transfected with LV‐circRbfox1 shRNA and a HuC overexpression plasmid, there was a significant reduction in both RBFOX1 mRNA and protein levels (Figure 5H,I). Importantly, it was demonstrated that the overexpression of HuC did not result in an increase in the expression of RBFOX1 in circumstances where circRbfox1 had been previously downregulated. Together, these data established that circRbfox1 may regulate the expression of RBFOX1 by modulating the subcellular localization of HuC.

### 3.7 RBFOX1 Contributes to Colonic Hypersensitivity by Regulating the Expression of Cav1.3 in Rats With Diabetes

**FIGURE 6:**
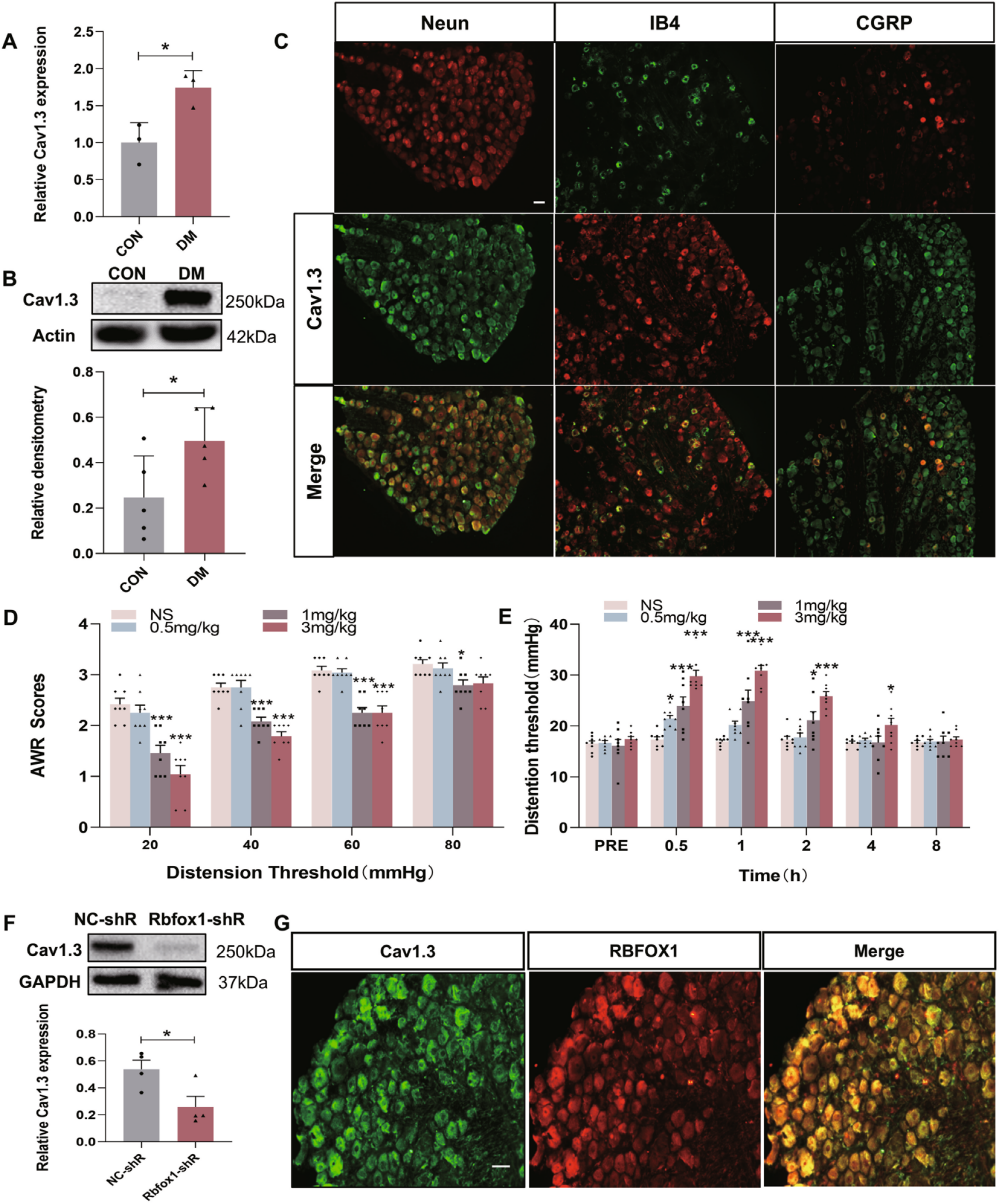
RBFOX1 contributes to colonic hypersensitivity by regulating the expression of Cav1.3 in rats with diabetes. (A) Cav1.3 mRNA level was greatly enhanced in diabetic rats compared with CON rats 4 weeks after injection (CON: *N* = 3, DM: *N* = 3, *p < 0.05, two sample *t*‐test). (B) Cav1.3 protein expression was greatly enhanced in diabetic rats compared with CON rats 4 weeks after injection (CON: *N* = 5, DM: *N* = 5, *p < 0.05, two sample *t*‐test). (C) Immunofluorescence results indicate co‐localization of Cav1.3 primarily with neurons labeled with NeuN, as well as with small to medium‐sized peptide‐neurons labeled with CGRP, and small to medium‐sized non‐peptidergic neurons labeled with IB4 in control rat of T13‐L2 DRGs. Scale bar = 50 μm. (D) AWR scores of diabetic rats after intraperitoneal injection of Isradipine at 0.5 h (NS: Normal saline, *n* = 8 for each group, *p < 0.05, ***p < 0.001, two‐way repeated‐measures ANOVA followed by Tukey post hoc test). (E) Intraperitoneal injection of Isradipine significantly alleviated colonic hypersensitivity in diabetic rats in a dose‐dependent manner and lasted for 4 h (NS: Normal saline, *n* = 8 for each group, *p < 0.05, ***p < 0.001, two‐way repeated‐measures ANOVA followed by Tukey post hoc test). (F) Cav1.3 protein expression was greatly decreased in Rbfox1‐shR treated diabetic rats compared with NC‐shRNA treated diabetic rats (Rbfox1‐shR: Rbfox1‐shRNA, NC‐shR: Negative control shRNA, *n* = 4 for each group, *p < 0.05, two sample *t*‐test). (G) The colocalization of Cav1.3 and RBFOX1 in T13‐L2 DRGs was detected using IF assay. Cav1.3 positive neurons are shown in green. RBFOX1 positive neurons are shown in red. Merge of double labeling of Cav1.3 and RBFOX1. Scale bar = 50 μm.

Our previewed studies have suggested some ion channels of DRG neurons play an important role in the generation of peripheral sensitization and nociceptive sensation [16]. Therefore, we detected the expression of multiple ion channels of T13‐L2 DRGs in diabetic rats and found that the mRNA and protein expression of L‐type calcium channel Cav1.3 was significantly increased (Figure 6A,B). Next, we performed immunofluorescence staining for localization of Cav1.3. As shown in Figure 6C, Cav1.3 was co‐localized in NEUN‐positive/CGRP‐positive/IB4‐positive neurons of T13‐L2 DRGs in control rats. We injected different concentrations of the L‐type calcium‐channel blocker Iradipine intraperitoneally at 4 weeks in rats with diabetes and found that the concentration of Iradipine at 1 mg/kg and 3 mg/kg significantly alleviated colonic hypersensitivity, as shown by a recording of AWR in response to CRD (Figure 6D,E). Furthermore, compared with LV‐NC shRNA, the Cav1.3 expression level was greatly decreased after a single intraperitoneal injection of LV‐Rbfox1 shRNA in rats with diabetes (Figure 6F). In addition, Cav1.3 and RBFOX1 were co‐expressed in T13‐L2 DRGs of control rats (Figure 6G). Thus, we proposed that RBFOX1 may contribute to colonic hypersensitivity by regulating the expression of Cav1.3 in rats with diabetes.

## 4 Discussion

This study elucidates a novel molecular pathway underpinning diabetic colonic hypersensitivity, centering on a previously uncharacterized circular RNA, circRbfox1. We demonstrate that circRbfox1 is significantly upregulated in T13‐L2 in rats with diabetes. Our data establish that this function of circRbfox1 is not as a miRNA sponge or a peptide template, but rather by directly interacting with the RNA‐binding protein HuC. This interaction is critical, as it facilitates the translocation of HuC from the cytoplasm to the nucleus. The nuclear accumulation of HuC, in turn, drives the increased transcription and expression of its target gene, Rbfox1, the parent gene from which circRbfox1 is derived. The ensuing upregulation of the RBFOX1 protein ultimately promotes colonic hypersensitivity by enhancing the expression of the L‐type calcium channel Cav1.3. Thus, we identify a coherent signaling axis—circRbfox1/HuC/RBFOX1/Cav1.3—that significantly contributes to the pathogenesis of diabetic visceral pain.

Our findings position circRNAs as key regulators in the complex pathophysiology of diabetic neuropathy, specifically in sensory dysfunction. While previous work implicated Circ_0000756 Mediates Oxidative Stress and Metabolic Memory in Diabetic Neuropathy [17], our study is the first to uncover a role for a circRNA in diabetic visceral pain, a common yet poorly understood clinical problem. circRNA can bind to the nuclear U1 ribonucleoprotein complex at the promoter region of the host gene, interact with RNA polymerase II, and promote the linear transcription of the host gene [18, 19]. Alternatively, they can bind to RNA‐binding proteins, interfering with their binding to the linear mRNA of the host gene, thus affecting the generation of the host gene's linear transcripts [20, 21]. The mechanism we describe is distinct and adds a new layer to the functional repertoire of circRNAs. By acting as a molecular chaperone that alters the subcellular localization of RBPs, circRbfox1 effectively amplifies the expression of its own host gene. This positive feedback loop, where a circRNA enhances the production of its linear counterpart, represents a sophisticated form of gene regulation that may be exploited in disease states. The persistence of the pain phenotype for at least four weeks after diabetes induction, and its reversal upon circRbfox1 knockdown, underscores its sustained and pivotal role in maintaining hypersensitivity, moving beyond a mere correlative observation to a causative factor.

The central role of the HuC translocation event is particularly noteworthy. Hu proteins are RNA‐binding proteins involved in diverse biological processes. There are four members of the Hu family: HuA, HuB, HuC, and HuD [22]. Hu proteins can modulate many post‐transcriptional aspects of RNA metabolism by binding to AU‐rich elements (AREs) or alternative polyadenylation sites [23]. Previous research has demonstrated the indispensable role of Hu proteins in orchestrating the differentiation, preservation, and vitality of neuronal, axonal, and synaptic structures [24]. The involvement of HuC, on the other hand, has been predominantly associated with paraneoplastic neurological syndromes and amyotrophic lateral sclerosis [25]. HuC is known to stabilize mRNAs and influence neuronal function [26]. Our data reveal that diabetic conditions subvert this normal function, leveraging the elevated circRbfox1 to sequester HuC in the nucleus. This redistribution is likely a key switch that reprograms the transcriptional landscape of DRG neurons, favoring a pro‐nociceptive state. The fact that HuC overexpression failed to rescue RBFOX1 expression when circRbfox1 was knocked down strongly suggests that circRbfox1 is not merely permissive but is the essential driver of HuC's nuclear shift and its subsequent transcriptional activity in this context.

The downstream effector of this pathway, Cav1.3, provides a plausible mechanistic link to neuronal hyperexcitability. L‐type calcium channels, including Cav1.3, are critical regulators of neuronal firing and synaptic transmission. Their upregulation in DRG neurons is a recognized mechanism underlying various chronic pain states [27, 28]. Several studies have indicated that RBFOX1 is an important regulatory component of L‐type calcium channels expression. L‐type calcium channels also affect pancreatic β‐cell function and insulin secretion, contributing to the development of diabetes and its complications [29]. Our results show that pharmacological blockade of these channels alleviates colonic hypersensitivity, and crucially, that their expression is controlled by the circRbfox1/HuC/RBFOX1 cascade. This connects the upstream epigenetic‐like regulation by a circRNA directly to the alteration of ion channel density and, consequently, to the aberrant sensory neuron activity that manifests as pain.

In conclusion, we have delineated a complete pathway from a specific circRNA to a functional pain outcome in diabetic colonic hypersensitivity. The circRbfox1/HuC/RBFOX1/Cav1.3 axis offers a novel conceptual framework for understanding how metabolic disturbances in diabetes can lead to persistent visceral pain through post‐transcriptional and transcriptional reprogramming of sensory neurons. Given the stability of circRNAs and their enrichment in the nervous system, circRbfox1 presents a promising and druggable target for developing much‐needed, non‐opioid therapeutics aimed at alleviating this painful diabetic neuropathy. Future studies exploring the presence and function of this pathway in human tissues will be essential to validate its clinical relevance.

## 5 Limitations

Although this study provides evidence for the involvement of the circRbfox1/HuC/RBFOX1/Cav1.3 axis in STZ‐induced visceral hypersensitivity, several limitations should be acknowledged. First, our findings were derived exclusively from a model of Type 1 Diabetes (T1D) using female rats. Given the well‐documented sexual dimorphism in pain processing and the prevalence of diabetic neuropathy in both sexes, it is crucial for future studies to investigate whether the identified mechanism operates similarly in male subjects. Second, diabetic neuropathic pain is a common complication in both T1D and Type 2 Diabetes (T2D), which may involve distinct yet overlapping pathological pathways. Therefore, the generalizability of the circRbfox1/HuC axis to T2D‐associated neuropathy remains to be established. Subsequent research using established models of T2D, such as *db/db* mice or high‐fat diet‐induced rodents, is warranted to explore the broader relevance of this axis across diabetic etiologies. Addressing these points will be essential for translating these mechanistic insights into a more comprehensive understanding of diabetic neuropathic pain and for developing universally effective therapeutic strategies.

## Author Contributions

S.Y.Z., Y.L. and C.D.N. performed the experiments, analyzed the data, and prepared figures and the manuscript. These authors contributed to this work equally. J.C, Y.J.L, S.H. and F.C.Z. performed the experiments and analyzed the data. J.H. revised the manuscript. H.‐H.Z. and G.‐Y.X. designed and supervised the experiments and finalized the manuscript. All authors contributed to the article and approved the submitted version.

## Funding

This work was supported by National Natural Science Foundation of China, 82071234, 82170836, 31400947. Noncommunicable Chronic Diseases‐ National Science and Technology Major Project, 2023ZD0507200. Gusu Talent Program, GSWS2022030. Jiangsu Youth Medical Talents Project, QNRC2016874. The project of the clinical research center of neurological disease of the Second Affiliated Hospital of Soochow University, ND2022B01. The Basic Frontier Innovation Cross Program of Suzhou Medical College, YXY2304059.

## Ethics Statement

All animal experiments were approved by the Institutional Animal Care and Use Committee at Soochow University (SYXK 2022–0043) and carried out in strict accordance with the International Association for the Study of Pain guidelines.

## Conflicts of Interest

The authors declare no conflicts of interest.

## Supporting information


**Figure S1:** Colonic hypersensitivity in STZ‐induced diabetic rats. (A) Compared with CON rats, the body weight exhibited a significant decrease in STZ‐induced diabetic rats. (*n* = 8 for both groups, ***p < 0.001, two‐way repeated‐measures ANOVA followed by Tukey post hoc test). (B) Compared with CON rats, the blood glucose levels were remarkably increased in STZ‐induced diabetic rats (*n* = 8 for both groups, ***p < 0.001, two‐way repeated‐measures ANOVA followed by Tukey post hoc test). (C, D) abdominal withdrawal reflex (AWR) was recorded as colonic sensitivity at 2 weeks and 4 weeks after injection of STZ. (E) CRD was used to grade the behavioral response of rats by assessing colonic hypersensitivity after injection of STZ. (*n* = 6 in each group, ***p < 0.001, two‐way repeated‐measures ANOVA followed by Tukey post hoc test).
The majority of STZ‐induced rats developed hyperglycemia and colonic hypersensitivity as shown by measuring the AWR in response to CRD.

## Data Availability

The data that support the findings of this study are available from the corresponding author upon reasonable request.
