# Supplementary figures and images for: CircRbfox1 Contributes to Colonic Hypersensitivity in Rats With Diabetes by Altering HuC Subcellular Localization to Regulate RBFOX1 Expression
Source: CNS Neurosci Ther. 2026 Apr 28;32(4):e70896. doi: 10.1002/cns.70896 (PMC13123447; doi:10.1002/cns.70896)

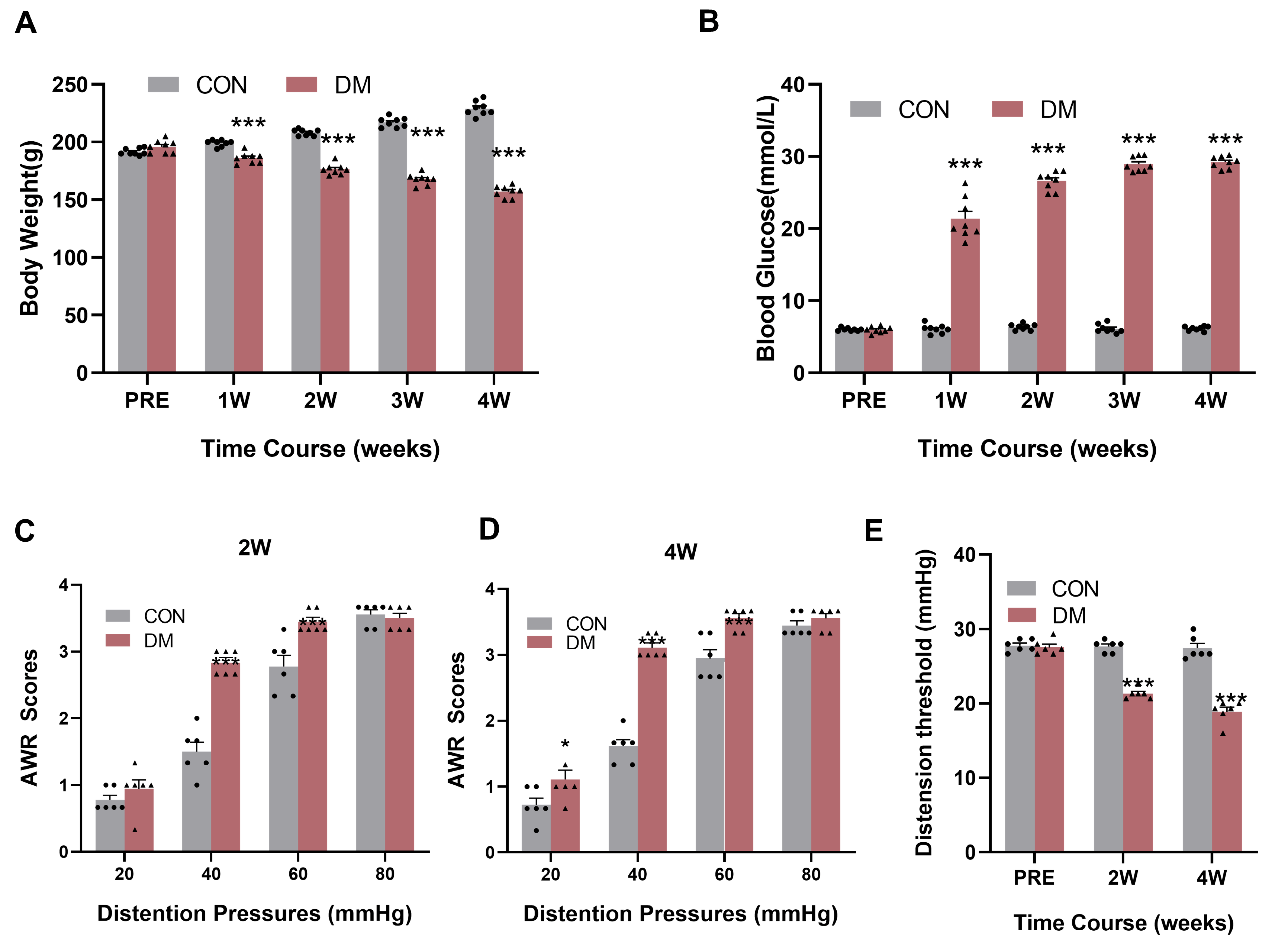

Supplement: Supplementary file 1 — Figure S1: Colonic hypersensitivity in STZ‐induced diabetic rats. (A) Compared with CON rats, the body weight exhibited a significant decrease in STZ‐induced diabetic rats. (n = 8 for both groups, ***p < 0.001, two‐way repeated‐measures ANOVA followed by Tukey post hoc test). (B) Compared with CON rats, the blood glucose levels were remarkably increased in STZ‐induced diabetic rats (n = 8 for both groups, ***p < 0.001, two‐way repeated‐measures ANOVA followed by Tukey post hoc test). (C, D) abdominal withdrawal reflex (AWR) was recorded as colonic sensitivity at 2 weeks and 4 weeks after injection of STZ. (E) CRD was used to grade the behavioral response of rats by assessing colonic hypersensitivity after injection of STZ. (n = 6 in each group, ***p < 0.001, two‐way repeated‐measures ANOVA followed by Tukey post hoc test). The majority of STZ‐induced rats developed hyperglycemia and colonic hypersensitivity as shown by measuring the AWR in response to CRD. [file CNS-32-e70896-s001.tif]
